# Crystal Structure of a Hidden Protein, YcaC, a Putative Cysteine Hydrolase from *Pseudomonas aeruginosa*, with and without an Acrylamide Adduct

**DOI:** 10.3390/ijms160715971

**Published:** 2015-07-14

**Authors:** Morten K. Grøftehauge, Daphne Truan, Adriana Vasil, Paul W. Denny, Michael L. Vasil, Ehmke Pohl

**Affiliations:** 1 School of Biological and Biomedical Sciences, Durham University, Durham DH1 3LE, UK; E-Mail: p.w.denny@durham.ac.uk; 2 Swiss Light Source, Paul Scherrer Institute, Villigen CH-5232, Switzerland; E-Mail: daphne.truan@a3.epfl.ch; 3 Department of Immunology and Microbiology, University of Colorado School of Medicine, Aurora, CO 80045, USA; E-Mails: adriana.vasil@ucdenver.edu (A.V.); mike.vasil@ucdenver.edu (M.L.V.); 4 School of Medicine, Pharmacy and Health, Durham University, Stockton-on-Tees TS17 6BH, UK; 5 Department of Chemistry, Durham University, Durham DH1 3LE, UK

**Keywords:** X-ray crystallography, micro-crystals, molecular replacement, YcaC, isochorismate family, protein octamer

## Abstract

As part of the ongoing effort to functionally and structurally characterize virulence factors in the opportunistic pathogen *Pseudomonas aeruginosa*, we determined the crystal structure of YcaC co-purified with the target protein at resolutions of 2.34 and 2.56 Å without *a priori* knowledge of the protein identity or experimental phases. The three-dimensional structure of YcaC adopts a well-known cysteine hydrolase fold with the putative active site residues conserved. The active site cysteine is covalently bound to propionamide in one crystal form, whereas the second form contains an *S*-mercaptocysteine. The precise biological function of YcaC is unknown; however, related prokaryotic proteins have functions in antibacterial resistance, siderophore production and NADH biosynthesis. Here, we show that YcaC is exceptionally well conserved across both bacterial and fungal species despite being non-ubiquitous. This suggests that whilst YcaC may not be part of an integral pathway, the function could confer a significant evolutionary advantage to microbial life.

## 1. Introduction

*Pseudomonas aeruginosa* infections are a major cause of morbidity and mortality in cystic fibrosis sufferers and in immunocompromised patients [1]. Treatment options are limited due to wide-ranging intrinsic and spontaneous antibiotic resistance [2]. Because of the significant clinical relevance of *P. aeruginosa*, a considerable amount of research has been directed towards this important opportunistic pathogen, but the functional and structural characterization of the complete set of gene products is, of course, far from complete. During our ongoing studies of *P. aeruginosa* virulence determinants, including the hemolytic phospholipase PlcH, which is secreted as the PlcHR2 heterodimer [3,4], we have crystallized and determined the structure of *P. aeruginosa* YcaC (PA1202 [5], UniProt Q9I4D). PlcHR2 is a major extracellular toxin that has been shown to be selectively toxic to human endothelial cells. It has been hypothesized that this property may be highly important to the mechanism that the pathogen uses to cause lesions and inhibit wound healing [3].

YcaC belongs to the isochorismatase-like protein family, which is a group of cysteine hydrolases known to work on vinyl ethers, conjugated primary amides and lactams. Of particular clinical relevance are the *Mycobacterium tuberculosis* nicotinamidase (also designated pyrazinamidase), which converts pyrazinamide into the active bacteriostatic/antibiotic pyrazinoic acid [6], and the *Streptomyces*/*Acinetobacter* streptothricin hydrolase, which inactivates the antibiotic streptothricin by hydrolyzing the lactam ring [7]. YcaC was co-purified with the target protein PlcHR2 during the expression of Se-methionine-substituted protein using a *P. aeruginosa* overexpression system. Micro-crystals were obtained during selenomethionine PlcHR2 complex crystallization experiments [4]. Crystal dimensions of less than 10 micrometers combined with low symmetry and limited resolution rendered anomalous data collection non-trivial, and consequently, phasing using single/multiple anomalous diffraction methods failed. The structure was finally solved by high-throughput molecular replacement methods. Each of the YcaC monomers contains 205 amino acids with a molecular weight of 22.5 kDa. The crystal structure of the monomer consists of a central six-strand parallel beta-sheet with alpha-helices on both sides and three C-terminal helices that interact with the neighboring monomer. The quaternary structure is an octameric cylinder, which is built from two stacked tetramers. The overall dimensions of the protein octamer in length and diameter are approximately 75 Å.

In order to further investigate the functional role of YcaC, we utilized bioinformatics analysis to characterize the evolutionary relationship between YcaC in both *E. coli* and *P. aeruginosa* and found that there is an unusually high sequence identity of >75%. Notably, the *ycaC* gene is also found in eukaryotic fungal genomes with a sequence identity of >50% to *Pseudomonas aeruginosa* YcaC. This high level of sequence conservation may indicate an important functional role across both prokaryotic and eukaryotic life.

## 2. Results and Discussion

### 2.1. Crystal Structure of YcaC from P. aeruginosa

**Figure 1:**
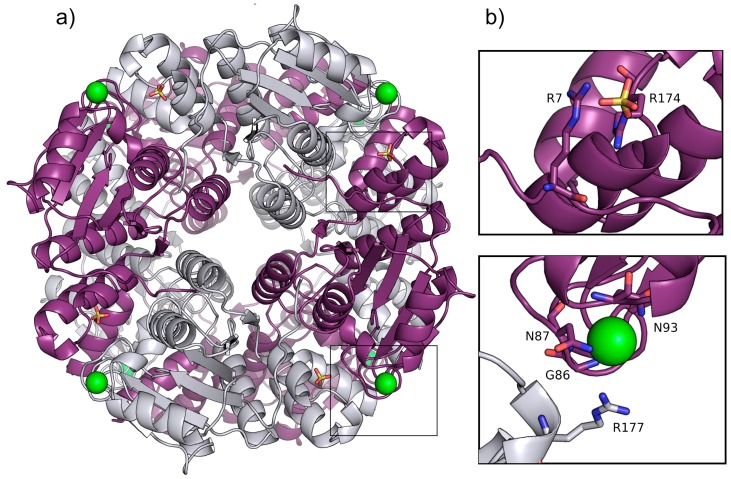
(**a**) Ribbon representation of *P.* *aeruginosa* YcaC octamer in crystal form I with chains alternately colored purple and silver, chloride as green spheres and sulfate as sticks with orange sulfur and red oxygens; (**b**) Close-up of the sulfate and chloride binding sites in chain A shown in purple with chain B depicted in silver. Stacked Arg 7 and 174 with the sulfate are shown in ball-and-stick representation. The chloride is shown with coordinating residues Gly86, Asn87 and Asn93 and the lid-forming Arg177 from chain B.

*P. aeruginosa* YcaC crystallized in two space groups with identical crystal morphology and similar resolution limits (Table 1). The quaternary structure is a back-to-back dimer of pin-wheel homotetramers, as shown in Figure 1a.

**Table 1:** Data collection and refinement statistics (numbers in brackets refer to the last resolution shell). The rather low R-factors relative to the resolution are likely due to the recent advances in protein X-ray crystallography and high non-crystallographic symmetry [8].

Parameter	Crystal Form I	Crystal Form II
Beamline	SLS X06SA	DLS I04
Wavelength (Å)	0.9537	0.9686
Resolution range (Å)	31.7–2.34 (2.42–2.34)	48.05–2.56 (2.66–2.56)
Space group	C2	P2_1_
Unit cell dimensions (Å), (˚)	158.36, 74.48, 141.06, β = 92.29	74.46, 114.53, 96.16 β = 91.9
No. of reflections	238,833 (3149)	387,741 (7803)
Unique reflections	60,390 (2522)	47,067 (2984)
Multiplicity	4.0 (1.3)	8.2 (2.6)
Completeness (%)	87 (36)	91 (58)
I/σ (I)	11.7 (4.6)	11.3 (2.6)
Wilson B-factor	12.1	26.5
*R*_merge_ (%)	15.6 (27.6)	21.4 (44.4)
CC1/2	0.979 (0.82)	0.99 (0.7)
*R*_work_ (%)	0.172 (0.224)	0.146 (0.224)
*R*_free_ (%)	0.204 (0.251)	0.191 (0.281)
No. of non-H-atoms	13,755	13,016
No. of protein residues	1616	1616
RMSD bond lengths	0.002	0.004
RMSD bond angles	0.7	0.8
Ramachandran plot favored (%)	98	98
Allowed (%)	1.9	2.3
Outliers	0.5	0
Average B-factor protein	14.0	27.9
Ligands	51.1	39.5
Solvent	21.5	28.3
PDB codes	4WGF	4WH0

The pin-wheel arrangement of YcaC appears to be vital to the function of the more closely-related enzymes, since each putative active site is made up of residues from one chain and the C-terminus of the neighboring chain. This C-terminal region from residue 165 and onwards contains three alpha-helices and a loop with what looks like a single-wind helix.

The YcaC monomer is 205 residues long; however, the *N*-formylmethionine appears to have been cleaved off, and residues 204 and 205 are disordered in our crystal structure. Residues 1–164 can be described as a Rossmann fold with six parallel beta-strands. There is a chloride ion coordinated by the main chain nitrogens of Gly86 and Asn87, the side chain nitrogens of Asn87 and Asn93 and the dipole moment of the helix 93–104 [9]; see Figure 1b. In a dynamic event in which the chloride was no longer positioned to stabilize the helix, the side chains of residues Asp92, Glu94 or Asp95 could potentially all adopt this position. Such a dynamic event could possibly allow substrate and product to enter and leave the active site. Arg177 of the neighboring chain does not appear to directly interact with the chloride, but could be forming a lid at a distance of 4–5 Å, discouraging the chloride from leaving. Furthermore, one sulfate ion per monomer is located at a salt bridging distance from the stacked guardinium head groups of Arg7 and Arg174. One additional sulfate is found bound to water molecules, Arg48 of chain F and Asn51 of chain D of a symmetry-related homo-octamer. The sulfate densities are absent in the crystal form II structure, which was crystallized with sodium malonate instead of ammonium sulfate.

Crystal form I also has one 2,5-hexanediol bound per sub-unit in the interface between subunits. The ligand density is poorly defined, and most of the interaction appears to be hydrophobic, but there are hydrogen bonds to residues Pro192, Asn196 or Glu71 of the neighboring chain. While the active site interaction between neighboring subunits is contained within the pin-wheel tetramer, the hexanediol binding bridges the dimerization of tetramers.

Both structures reported adopt virtually the same conformation as evidenced by an RMSD between all *C*-alpha atoms of the A chains of crystal forms I and II of 0.19 Å. The RMSDs between chain A of YcaC with all other independent chains in both crystal forms I and II lie in the range of 0.3–0.4 Å. These values are well within the experimental error of crystal structure determinations at this resolution.

**Figure 2:**
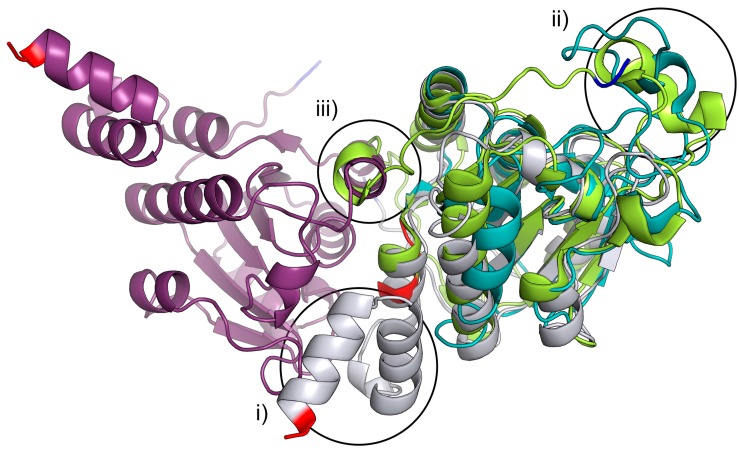
Ribbon representation of the least-squares superposition of chain A and B of Pa YcaC shown in purple and silver, the isochorismatase PhzD from *P.* *aeruginosa* (1NF9) [11] in lime and Mtb PncA (3PL1-a nicotinamidase) [12] in teal. N-terminal residues are colored blue, and C-terminal residues are colored red. (i) PhzD and PncA are both missing the YcaC C-terminal helices that form the active site with the neighboring chain; (ii) the large loop would prevent tetramerization if present in YcaC; (iii) as would the additional N-terminal helix present in PhzD. N- and C-termini are highlighted in blue and red, respectively.

It is interesting to note that as in this case, the homologous *E. coli* YcaC was also crystallized as a contaminant, but of *E. coli* l-glutamic acid decarboxylase. Previous crystal structure determinations of related proteins have shown that the pin-wheel homotetramer arrangement is also found in protozoan homologues from *T. cruzi* (1YZV), *L. donovani* (1X9G) and *L. major* (1XN4) [10]. In the structures of these eukaryotic homologues, the third helix is replaced by a shorter stretch of residues without a secondary structure. However, this arrangement is not found in other members of the isochorismatase-family for which the structures have been determined. The isochorismatase PhzD from *P. aeruginosa* [11], as well as the nicotinamidase PncA from *M. tuberculosis* [12] are missing the C-terminal helix, which is part of the active site in the YcaC structure presented here (Figure 2i). In addition, the extended loop, as well as the additional N-terminal helices (Figure 2ii,iii) prevent tetramerization.

### 2.2. Active Site and a Potential Reaction Mechanism

The active site is formed in the interface between individual chains of the pin-wheel homotetramer. In the crystal form II structure, the active site Cys118 is covalently modified to form an *S*-mercaptocysteine, while in the crystal form I structure, Cys118 is linked to a propanamide. Most likely, the reducing agent used in the crystallization, tris(2-carboxyethylphosphine) (TCEP), acted as a catalyst for the phosphine-catalyzed addition of excess acrylamide from the preparative native gel electrophoresis to the nucleophilic thiol of Cys118 [13]. Acrylamide is sometimes detected via mass spectrometry as an adduct to cysteines of proteins that have been run through an SDS-PAGE and have been reported twice before in X-ray crystallographic protein structures (PDB Codes 4GYL [14] and 3ZVI [15]). Cys170 was not similarly modified despite also pointing to the active site. There is no solvent access to the active site cavity in the structure, which suggests a highly dynamic enzyme, opening and closing to move substrate and product in and out of the active site.

The propanamide has a hydrogen bond to Ser59 at a distance of 3.0–3.1 Å, which supports the idea that acylamide is an accidental suicide substrate for a nucleophilic thiol. Asp19 is perfectly positioned to extract a proton from Cys118 and to activate the nucleophile. In addition, protonation of Asp19 would disrupt the salt bridge to Arg84, which, in turn, could change the conformation of the chloride-binding loop 84–94 and allow access to and from the active site. The nearby residue Phe60 is in an energetically-unfavorable rotamer, while orthogonal to Trp176 of the next chain, and this strained interaction could also be part of the opening mechanism. Greater restraints on rotamer conformations result in Phe60 no longer being an outlier, but instead produces a steric clash with well-defined water molecules. There is a *cis*-peptide bond between Val113 and Val114 in both structures, and the *cis*-peptide is conserved in the structure of YcaC from *E. coli*; the amide is pointing to the active site and is very close to Cys118. We do not see any evidence of a water molecule that could be used in a hydrolysis reaction. Apart from Leu187 being replaced by a phenylalanine, the active site is completely conserved between *P. aeruginosa* and *E. coli*. Notably and in contrast, in the protozoan proteins, the active site is solvent-accessible. Although Cys118 and Asp19 are completely conserved, Arg84 is replaced by a lysine, and a tyrosine is found in place of Phe60. Finally, the side chain of Trp176 from the neighboring monomer is replaced by a more distant phenylalanine.

In summary, there are enough differences in the active site to assume that the protozoan and prokaryotic enzymes could work on different substrates.

### 2.3. Sequence Conservation and Evolutionary Origin

YcaC from *E. coli* and *P. aeruginosa* is unusually well-conserved with a sequence identity of approximately 77%, compared to *P. aeruginosa* PhzD and *E. coli* EntB, iso-chromatases known to be active on the same substrates, which share a sequence identity of only 46% (Table 2). Furthermore, as well as being well conserved in bacterial species, YcaC orthologues with identities higher than 50% are also found in a number of fungal species. Performing a BLAST search on the UniProt database also returned sequences in *Populus trichocarpa* (Western balsam poplar) and *Ceratitis capitata* (Mediterranean fruit fly). These sequences demonstrated greater similarity to bacterial YcaC than to the fungal orthologues, indicating that there may be no common eukaryotic origin for the gene.

Table 2 illustrates the sequence identity relationship between isochorismatase family proteins in *E. coli* and *P. aeruginosa*. Whilst sequence identity is a poor tool for ranking relatedness at values of less than 30%–35%, it is clear that YcaC has an unusually high level of sequence conservation for a protein of this family.

**Table 2:** Sequence identity matrix of YcaC and paralogues in *P. aeruginosa* (Pa) and *E. coli*. (Ec). PncA is a nicotinamide hydrolase; PhzD and EntB are isochorismatases; RutB is a peroxyureidoacrylate/ureidoacrylate amidohydrolase; YecD, like YcaC, is of unknown function. Pa1–5 are *P. aeruginosa* paralogues of YcaC; Pa2 is found in strain PA7, but not in PAO1. The Pa plasmid is an isochorismatase family protein found on a virulence plasmid that confers antibiotic and heavy-metal resistance, isolated from a clinical strain of *P. aeruginosa*. UniProt Accession Numbers: Pa YcaC, Q9I4D6; Ec YcaC, P21367; Ec PncA, P21369; Pa PhzD, Q7DC80; Ec EntB, P0ADI4; Ec RutB, P75897; Ec YecD, P0ADI7; Pa1, Q9I162; Pa2, A6V5N9; Pa3, Q9HX63; Pa4, Q9HXL2; Pa5, Q9I348; Pa plasmid, G8CP33. Highly significant sequence identities (>30) are highlighted in bold.

Sequence	Ec YcaC	Ec PncA	Pa PhzD	Ec EntB	Ec RutB	Ec YecD	Pa1	Pa2	Pa3	Pa4	Pa5	Pa Plasmid
Pa YcaC	**76.6**	25.0	19.8	16.0	16.9	18.4	**45.1**	**38.0**	22.6	15.2	18.6	23.8
Ec YcaC		24.2	21.0	17.3	17.5	17.2	**44.4**	**38.4**	23.3	16.3	17.4	19.2
Ec PncA			21.4	22.1	28.8	23.2	21.8	22.8	23.9	28.1	26.5	**32.3**
Pa PhzD				**46.1**	23.8	11.3	17.5	17.9	24.4	21.3	18.7	17.3
Ec EntB					22.4	20.8	15.7	12.0	23.2	18.2	20.9	20.5
Ec RutB						25.3	15.8	16.7	22.4	27.9	26.1	26.2
Ec YecF							11.3	20.6	26.1	27.0	26.0	27.0
Pa 1								**33.8**	18.4	15.8	19.9	20.0
Pa 2									16.0	17.7	16.7	23.0
Pa 3										26.7	**34.7**	27.6
Pa 4											**32.7**	**31.5**
Pa 5												**30.4**

**Figure 3:**
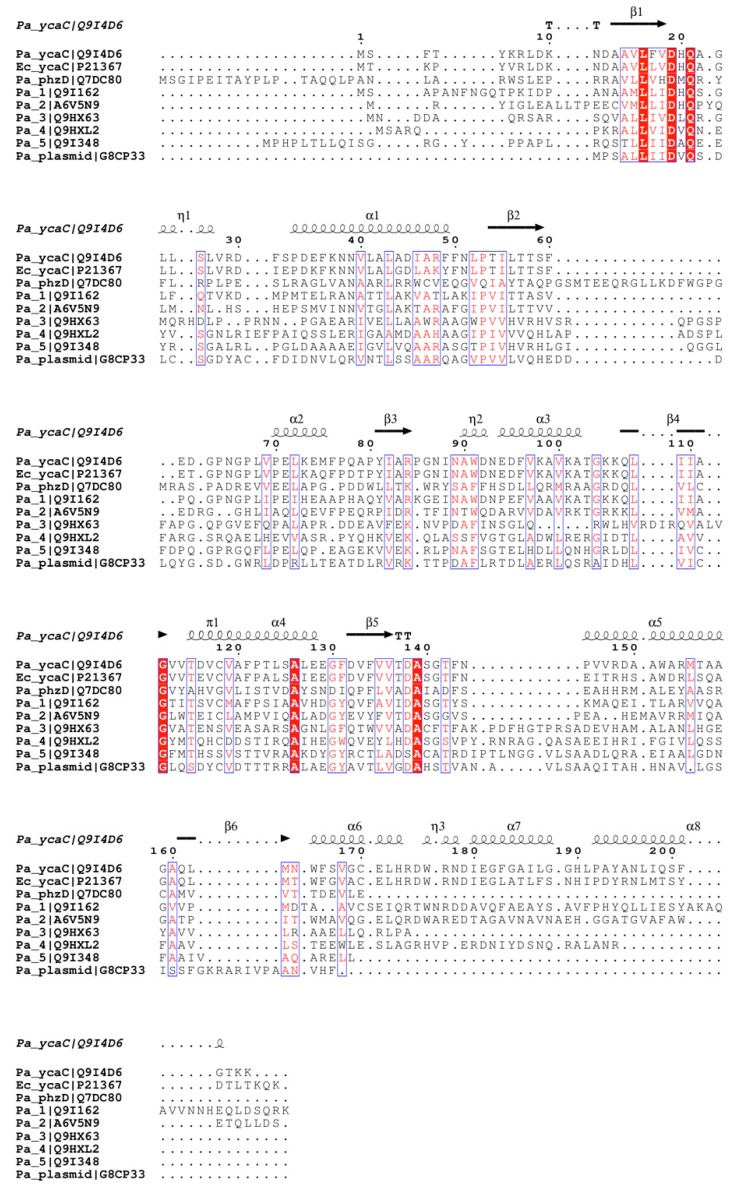
Sequence alignment of YcaC homologues with secondary structural elements annotated based on the Pa YcaC crystal structure presented here. Conserved residues are highlighted in red (highly conserved) and white on red background (absolutely conserved).

Based on the multiple sequence alignment given in Figure 3, it appears that two more isochorismatase-like proteins in *P. aeruginosa* form tetramers, and possibly octamers, considering that the three C-terminal helices are conserved in Pa 1 (UniProt Q9I162) and Pa 2 (UniProt A6V5N9).

## 3. Experimental Section

Protein expression, purification and crystallization: YcaC was inadvertently co-purified with the heterodimer *P. aeruginosa* PlcHR2 when overexpressed in *P. aeruginosa* PADD1976 [16], with the *metZ* gene inactivated by mutation, using a T7 expression plasmid (pADD3268). The bacteria were grown in minimal media supplemented with 50 μg/mL l-selenomethionine (SeMet). YcaC was not detected in the protein preparation via SDS-PAGE or mass spectrometry, as previously reported [4], and no YcaC crystals were produced from native PlcHR preparations from *P. aeruginosa* with an intact *metZ* gene grown in minimal M9 media. Protein production and crystallization in space group C2 (crystal form I) have been described previously [4]. Briefly, cells were grown at 37 °C, induced with IPTG at OD_590_ ≈ 0.6 and grown for another 12 h at 32 °C. Cells were pelleted by centrifugation at 10,000× *g* and then discarded. The supernatant was diluted with cold ddH_2_O to 2.5-times the original volume. Batch anion exchange chromatography was performed by the addition of pre-equilibrated micro-granular diethylaminoethyl cellulose DE52, 30 min of stirring in a cold room and washing and elution with 500 mM NaCl in a funnel. The eluate was concentrated and dialyzed before size exclusion chromatography by continuous elution preparative native gel electrophoresis on a Bio–Rad Model 491 Prep Cell. Fractions were selected by testing the activity in a p-nitrophenyl phosphocholine assay [17] and comparing with peaks in the chromatogram.

Crystal form I crystals were obtained with a protein solution of 9 mg/mL SeMet PlcHR2 in 50 mM NaCl, 50 mM Tris–HCl, pH 7.4, and 0.1 mM TCEP, a reservoir solution of 26% *w*/*v* PEG 3350, 400 mM (NH_4_)_2_SO_4_ and 70 mM bis–Tris–HCl pH 6.75 and a 40% *v*/*v* 2,5-hexanediol additive in sitting drops with a drop ratio of 2 μL protein, 0.8 μL reservoir and 0.2 μL additive. The protein solution was filtered and ultra-centrifuged before setting up drops to reduce nucleation, and crystals were obtained from seeding with microcrystals. Crystals of maximal dimensions 100 μm × 5 μm × 5 μm appeared after a month and were harvested after two months.

Crystal form II crystals were obtained with a protein solution of 5.3 mg/mL SeMet PlcHR2 in 50 mM NaCl, 5 mM MgCl_2_, 50 mM Tris–HCl pH 7.4 and 0.1 mM TCEP and a reservoir solution of 20% PEG 3350, 200 mM Na malonate and 100 mM MES–NaOH, pH 6.75. Using an Innovadyne 96+8 Xtal, sitting drops were set up with 200 nL protein solution and 100 nL reservoir solution. Crystals appeared after more than 3 months and grew to maximal dimensions of 100 μm × 10 μm × 10 μm. In both cases, crystals were too small and fragile for mass spectrometric analysis, but sufficiently large for micro-focus X-ray data collection on third generation synchrotron sources [18].

Data collection and processing: The large length-to-width ratio both meant that crystals broke exceptionally easily and that they could only be fished with mylar loops. Inclined MicroLoops E from MiTeGen (Ithaca, NY, USA) were used, but the crystals adhered to the mylar rather than fitting in the inclined aperture. In addition, many of the same challenges seen for micro-crystals were encountered, such as short life span in the X-ray beam [19]. For crystal form I, data were collected at the Swiss Light Source, beamline X06SA equipped with a CCD detector (Marresearch, Norderstedt, Germany), at 100 K in 12 wedges of 20° images each along the length of the crystal. Anomalous data referring the collection of Friedel pairs were incomplete due to the low crystallographic symmetry combined with noticeable radiation decay. Data were processed with XDS [20] and scaled with AIMLESS [21] through the Xia2 [22,23,24] command-line interface using the option to use all images for indexing. The needle crystal morphology was reflected in the anisotropy of the diffraction limit and resulted in low completeness and multiplicity in the highest resolution shell, but the low anisotropy of the diffraction spots themselves resulted in acceptable *R*_merge_ and *I*/sigma. An additional 13th wedge of only 10 images was also collected, but could not be scaled with the rest of the data. We have previously reported a dataset collected from the same crystallization and beamline, but with a lower resolution [4]. This dataset was collected from a single point in the crystal. Using the Xia2 pipeline enabled the scaling of many datasets.

Crystal form II data were collected at Diamond Light Source, beamline I04 equipped with a Pilatus Pixel detector [25], at 100 K as a line diffraction experiment. Images were collected over 9 wedges with each wedge consisting of 40 0.5° images to cover 0–180 degrees, followed by the same collection strategy twice to cover 180–360 degrees. As in crystal form I, no significant anomalous signal was detected, presumably due to limited redundancy in this low symmetry monoclinic crystal form.

Structural determination: The poor anomalous signal meant that the selenomethionine sites could not be determined in any of the available software packages using anomalous data. The phases were determined by submitting the reprocessed crystal form I data for Wide Search Molecular Replacement (WSMR) at the SBGrid webpage [26]. Ninety-nine thousand two-hundred five structures truncated to their Structural Classification of Proteins (SCOP) domain definitions were used for molecular replacement in Phaser [27] using the Open Science Grid computing resources. Two hits scored a *Log-Likelihood-Gain* (LLG) of 63 and TFZ scores of 10.6 and 10.7, respectively, with 10%–20% coverage of the expected asymmetric unit contents. The two hits were the A and B chain of YcaC from *E. coli* (PDB Entry 1YAC) [28], which consists of a central beta-sheet with alpha-helices on both sides, as predicted for the larger domain in Pa PlcH. In our hands, TFZ and LLG were 10.7 and 246 when searching for one component and 24.6 and 843 when searching for two components with variance set to 30% sequence identity (because SBGrid WSMR uses 30% identity). We had reprocessed the data with updated versions of AIMLESS and Xia2 and used a more recent version of Phaser, which may account for the variance in the LLG score. Density modification and solvent flattening approaches with and without auto-tracing or autobuild failed to produce an interpretable map. Further inspection of 1YAC revealed that the same packing was present in both the 1YAC and the molecular replacement solutions, and that 1YAC was an octamer, which fit with the size of the asymmetric unit. TFZ was 24.6 and LLG 13,781 when searching for eight components with variance set to 30% sequence identity. Searching for 8 components in our crystal form II dataset gave TFZ of 63 and LLG of 11,415.

The structure was refined using Phenix.refine [29], and amino acids were mutated in Coot [30], where the side chain densities clearly did not match the sequence. The sequence for the ortholog of *E. coli* YcaC in *P. aeruginosa* strain PAO1 (PA1202) was then used, and the side chain substitutions were found to be correct, barring a few missing changes. Phenix.autobuild was then run using the refined and edited structure, the correct sequence and the crystal form I data. The autobuild results were used for molecular replacement in the crystal form II data, and that structure was then rebuilt using Phenix.autobuild. Both structures were refined and remodeled using Phenix.refine and Coot. When the structures could no longer be improved, they were submitted to the PDB_REDO server. PDB_REDO resulted in lower R-factors and fewer rotamer outliers, but with higher bond distance and bond angle RMSD. After PDB-REDO, rebuilding with Coot and Phenix.refine, all statistics were however improved or the same in all categories. Final refinement in Phenix used xyz positions, individual B-factors TLS-movements, non-crystallographic symmetry (NCS) torsional-angle restraints and optimization of weights. Final *R*_work_/*R*_free_ were 17.0%/20.7% for crystal form I and 14.8%/19.1% for crystal form II; full statistics are given in Table 1. All crystallographic data have been deposited at the Protein Data Bank [31] under Accession Codes 4WH0 and 4WGF, respectively.

Bioinformatic analysis: Sequences for the sequence identity matrix in Supplemental Table I were found by first using the HHPred web server [32] with the Pa YcaC sequence to identify proteins with a similar fold for which structures were available. Twenty-seven hits had a probability score of 100, and sequences from each of them were run through the NCBI DELTA-BLAST [33] tool against *E. coli* and *P. aeruginosa* genomes. Protein sequences with a sequence identity ≥20% and an *E*-value ≤ 10^−34^ were collected. Sequences from strains K12 and PAO1 were used where available, and one sequence from a *P. aeruginosa* virulence plasmid was also included. After discarding redundant sequences, the resulting 13 sequences were aligned using T-Coffee Expresso, since large amounts of homologous structural information were available [34]. The resulting multiple sequence alignment was reformatted in T-Coffee with the command “t_coffee-other_pg seq_reformat-in<msa>-output sim” to yield the identity values. RMSD values were calculated only on chain A with SuperPose [35] from the CCP4 package [24].

## 4. Conclusions

In determining the crystal structure of *Pseudomonas aeruginosa* YcaC, we identified elements specific for the quaternary structure arrangement and recognized them in paralogs of YcaC in *Pseudomonas*. We have ascertained that the putative catalytic residue is indeed nucleophilic and reacts with electrophiles. The fact that it was only co-purified for us when producing the seleno-methionine-substituted protein preparation suggests a function in stress response or nutrient metabolism. If YcaC is only expressed under stress and performs a single hydrolysis reaction, then that could also explain why the precise function of YcaC has yet to be determined. Both *P. aeruginosa* and *E. coli* YcaC were crystallized as contaminants of other protein preparations [28], which could indicate a privileged structure for crystallization at very low concentrations.

We found two ligand binding sites per monomer, and one of these sites contains a highly nucleophilic thiol. If, like other isochorismate-like bacterial proteins, YcaC is involved in antimicrobial drug resistance, the high propensity of YcaC to crystallize along with the enveloped ligand binding sites may make it an interesting target for structure-based drug design. The lack of ubiquity across bacterial pathogens means that any YcaC-inhibiting compounds could be narrow-spectrum antibiotics. This lack of ubiquity, coupled with the high level of conservation, suggests horizontal gene transfer as a means of acquisition for both bacteria and fungi and indicates that YcaC confers a significant selective advantage.
